# Health financing in the African Region: 2000–2009 data analysis

**DOI:** 10.1186/1755-7682-6-10

**Published:** 2013-03-06

**Authors:** Luis Gomes Sambo, Joses Muthuri Kirigia, Juliet Nabyonga Orem

**Affiliations:** 1 World Health Organization, Regional Office for Africa, Brazzaville, Congo; 2 World Health Organization, Health Systems and Services Cluster, Country Office, Kampala, Uganda

## Abstract

**Background:**

In order to raise African countries probability of achieving the United Nations Millennium Development Goals by 2015, there is need to increase and more efficiently use domestic and external funding to strengthen health systems infrastructure in order to ensure universal access to quality health care. The objective of this paper is to examine the changes that have occurred in African countries on health financing, taking into account the main sources of funding over the period 2000 to 2009.

**Methods:**

Our analysis is based on the National Health Accounts (NHA) data for the 46 countries of the WHO African Region. The data were obtained from the WHO World Health Statistics Report 2012. Data for Zimbabwe was not available. The analysis was done using Excel software.

**Results:**

Between 2000 and 2009, number of countries spending less than 5% of their GDP on health decreased from 24 to 17; government spending on health as a percentage of total health expenditure increased in 31 countries and decreased in 13 countries; number of countries allocating at least 15% of national budgets on health increased from 2 to 4; number of countries partially financing health through social security increased from 19 to 21; number of countries where private spending was 50% and above of total health expenditure decreased from 29 (64%) to 23 (51%); over 70% of private expenditure on health came from household out-of-pocket payments (OOPS) in 32 (71%) countries and in 27 (60%) countries; number of countries with private prepaid plans increased from 29 to 31; number of countries financing more than 20% of their total health expenditure from external sources increased from 14 to 19; number of countries achieving the Commission for Macroeconomics and Health recommendation of spending at least US$34 per person per year increased from 11 to 29; number of countries achieving the International Taskforce on Innovative Financing recommendation of spending at least US$44 per person per year increased from 11 to 24; average per capita total expenditure on health increased from US$35 to US$82; and average per capita government expenditure on health grew from US$ 15 to US$ 41.

**Conclusion:**

Whilst the African Region (AFR) average government expenditure on health as a per cent of THE increased by 5.4 per cent, the average private health expenditure decreased by the same percentage between 2000 and 2009. The regional average OOPS as a per cent of private expenditure on health increased by 4.9 per cent. The average external resources for health as a percentage of THE increased by 3.7 per cent. Even though on average the quantity of health funds have increased, we cannot judge from the current study the extent to which financial risk protection, equity and efficiency has progressed or regressed.

In 2009 OOPS made up over 20% of total expenditure on health in 34 countries. Evidence shows that where OOPS as a percentage of total health expenditure is less than 20%, the risk of catastrophic expenditure is negligible. Therefore, there is urgent need for countries to develop health policies that address inequities and health financing models that optimize the use of health resources and strengthen health infrastructure. Increased coverage of prepaid health-financing mechanisms would reduce over-reliance on potentially catastrophic and impoverishing out-of-pocket payments.

## Background

In the 2000 United Nations Millennium Declaration, UN Member States pledged to meet three health Millennium Development Goals (MDGs): reduce child mortality; improve maternal health; and combat HIV/AIDS, malaria and other diseases [1]. In the 2001 Abuja Declaration [2], Africa Heads of State committed themselves to take all necessary measures to ensure that the needed resources are made available from all sources and that they are efficiently and effectively utilized. In addition, they pledged to set a target of allocating at least 15% of their annual national budget to the improvement of the health sector.

The African Heads of State urged developed countries to fulfil their commitment of allocating 0.7% of their gross national product (GNP) as official development assistance to developing countries. In 2002, in Paragraph 42 of the Monterrey Consensus, world leaders reiterated their commitment, stating that “we urge developed countries that have not done so to make concrete efforts towards the target of 0.7 per cent of gross national product (GNP) as ODA to developing countries” [3] p.14.

It is critically important for African countries to increase health budget allocations if they want to move towards improved health status of their people and achieve international health goals. This is consistent with the Abuja commitment and should be advocated for various reasons. First, health is a prerequisite for economic growth and human development [4]. Second, health is a human right which every citizen is entitled to [5]. Third, national health systems are underfunded and require additional investments to boost capacities to deliver essential cost effective interventions [6-8]. Four, external aid cannot replace the required national efforts and resources that should be invested to guarantee peoples’ health.

The most compelling argument for striving to meet (and even exceed) the Abuja 15% target is that most countries in the African Region are not on track to achieving the health MDGs and some indicators such as the under-five mortality rate and maternal mortality ratio are disproportionately too high compared to other regions. The 2010 average under-five mortality rate for the African region (AFR) of 119 per 1000 live births is higher than that of the region of the Americas (AMR) by 6.6 fold/times, the European region (EUR) by 8.5 times, Eastern Mediterranean region (EMR) by 1.8 times, South Eastern Asia Region (SEAR) by 2.1 times, and Western Pacific region (WPR) by 6.3 times. Average maternal mortality ratio for the African region of 480 per 1000 live births is higher than that of the Americas by 7.6 fold/times, the European region by 24 times, Eastern Mediterranean region by 1.9 times, South Eastern Asia Region by 2.4 times, and Western Pacific region by 9.8 times [9].

The objective of this paper is to examine the changes that have occurred in African countries on health financing, taking into account the main sources of funding over the period 2000 to 2009.

## Methods

National health expenditure encompasses all expenditures for activities whose primary purpose is to restore, improve and maintain health for the nation and for individuals during a defined period of time [10]. National health account (NHA) is a tool for systematic, comprehensive, and consistent monitoring of resource flows in a country’s health system. Specifically, the NHA tracks the flow of health system resources from financing sources (i.e. entities that provide the funds), financing agents (entities that receive and use funds to pay for health activities), providers (entities that receive money to produce health activities), functions (types of public health goods and services provided) and health system inputs (e.g., health workforce, medical products and technologies, health facilities, vehicles, utilities) to beneficiaries [10].

The total health expenditures consist of public funds, private funds and donor funds. Public funds consist of mainly funds from central government revenue, regional and municipal government revenue and return on assets held by a public entity. The private funds compose of essentially employer funds, household funds and funds from non-profit institutions serving individuals. The rest of the world funds (or donor funds) include bilateral grants, multilateral international grants and funds from funds contributed by institutions (including foundations) and individuals (diaspora) outside the country.

### Data

WHO African Region has a total of 46 countries. The NHA data on all countries (except for Zimbabwe whose data was not available) were obtained from the World Health Statistics 2012 report [11]. It consisted of levels of total and government per capita expenditure on health, total expenditure on health (THE) as a percentage of gross domestic product (GDP), general government expenditure on health as a percentage of total expenditure on health, private expenditure on health (PrTHE) as a percentage of total expenditure on health, general government expenditure on health as a percentage of total government expenditure, external (donor) expenditure as a percentage of total expenditure on health, social security expenditure on health as a percentage of general government expenditure on health, out-of-pocket expenditure as a percentage of private expenditure on health, and private prepaid plans as a percentage of private expenditure on health. In this study we have attempted to compare the NHA data for 2000 with that of 2009. The analysis was done using Excel software.

## Results and discussion

### Percentage of GDP spent on health

**Figure 1:**
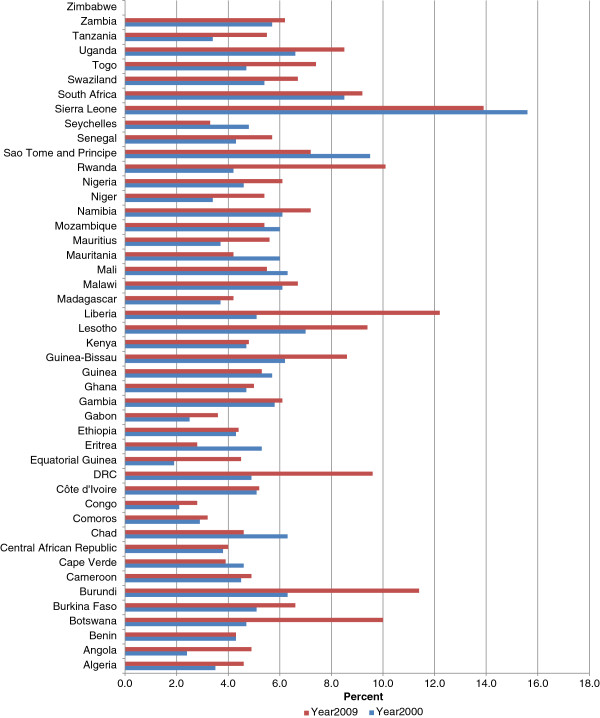
Total health expenditure as % of gross domestic product.

Figure 1 shows total health expenditure as a percentage of GDP. In 2000, 24 countries spent less than 5% of their GDP on health; 20 countries spent between 5% and 10%; and 1 country spent over 10%. In 2009, seventeen countries spent less than 5% of their GDP on health; 24 countries spent between 5.0 and 10%; and remaining 4 countries spent above 10%. The percentage of countries spending less than 5% of their GDP on health declined from 53% in 2000 to 38% in 2009. The percentage of GDP spent on health increased in 34 countries; decreased in 10 countries; and remained constant in one country. The average percentage of GDP spent on health in the African Region increased slightly from 5.5 in year 2000 to 6.5 in year 2009.

In 2009, the WHO Region of the Americas (AMR) on average spent the highest percentage of GDP on health of 14.4% compared to 9.3% in the European Region (EUR), 6.5% in both the African Region (AFR) and the Western Pacific Region (WPR), 4.7% in the Eastern Mediterranean Region (EMR), and 3.8% in South-Eastern Asia Region (SEAR).

The reader will recall that GDP is the market value of all final goods and services officially made within the borders of a country in a year. GDP is a sum of consumption, investment, government spending and net exports (exports minus imports). Therefore, an increase in any one of those variables, holding others constant, spontaneously expands the size of GDP and vice versa. Intuitively, as the absolute size of GDP increases, the proportion spent on health would be expected to increase. For example, a recent study estimated that between 2008 and 2009, the GDP of the African region decreased by US$94 billion due to the on-going global financial and economic crises [12]. The authors speculated that that decrease is likely to be reflected in reductions in expenditures on health. The size of GDP allocated to health sector hinges mainly on priority each country attaches to health development and on the rate of economic growth.

### Government funds

#### Government expenditure on health as a percentage of total expenditure on health

**Figure 2:**
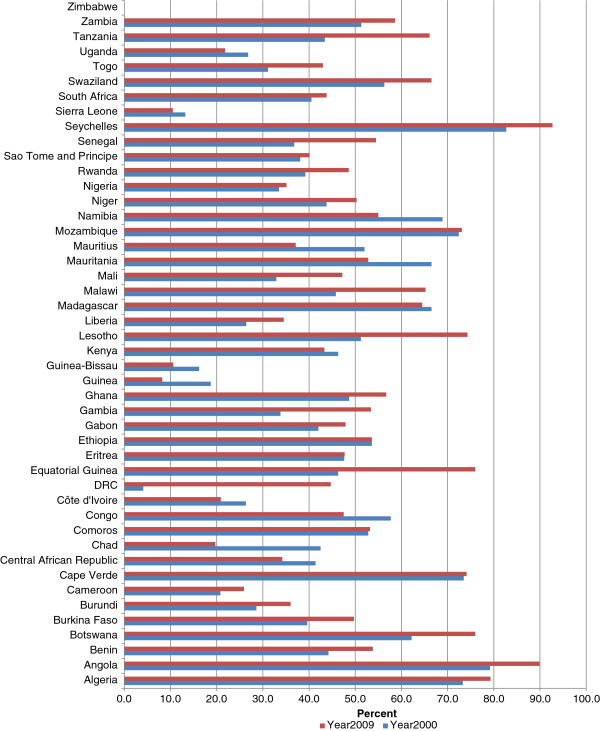
General government expenditure on health as % of total expenditure on health.

General government expenditure on health includes health expenditure at all levels (and ministries) of government, including the expenditure of public corporations. Figure 2 depicts the general government expenditure on health as a percentage of total expenditure on health (THE). In 2000, the general government expenditure as a percentage of THE was less than 30% in 9 countries, 30-60% in 27 countries, and over 60% in in 9 countries. In 2009, general government expenditure on health made up less than 30% of total health expenditure in 7 countries; 30-60% in 26 countries; and over 60% in 12 countries. In 2000, over 50% of the total expenditure on health in 16 countries was from government sources, compared to 22 countries in 2009. Government spending on health as a percentage of THE increased in 31 countries and decreased in 13 countries between years 2000 and 2009.

The AFR average government expenditure on health as a per cent of THE was 43.9% in 2000 compared to 49.3% in 2009. In 2009, the percentage of THE from government sources was 74.9% in EUR, 64.4% in WPR, 50.9% in EMR, 49.3% in AMR, 43.9% in AFR, and 37.1% in SEAR.

The government is an important source of health financing for most of the countries in the world. Why? Governments’ involvement in health financing might be justified for a number of reasons. Firstly, as a provider of “public goods”, that is, goods whose use by one person does not reduce its availability for use by other people. One can think of various health interventions with public goods characteristics. (i) Control of *schistosomiasis* through vector control measures such as mollusciciding reduces the risks of infection for all people living in the vicinity. (ii) Provision of clean water and sanitation reduces the risk of cholera outbreak in prone areas; which benefits not only people living in those areas but also tourists. (iii) Eradication of smallpox has enabled the world to enjoy environment free of smallpox without having to bear the cost. (iv) All people benefit directly or indirectly from epidemiological surveillance and information about outbreaks of infectious diseases since it facilitates government’s preparedness and response to epidemic-prone diseases [13]. The main properties of public goods, such as enjoyment of smallpox free environment, are “non-rivalrous” in consumption (ability of all people to benefit from a good once it is produced) and non-excludability (inability to exclude any individual or group from the benefits) [14].

Secondly, to deal with “externalities or spill-over effects”, i.e. the costs or benefits imposed on people not directly involved in a market exchange. An example of external cost is when a person smokes next to a non-smoker, which leads the latter to passive smoking increasing the risk of disease and related health care costs. Another example is when a HIV infected person has unprotected sexual intercourse with a person who is HIV-negative. This exposes the latter to risk of infection and risk of incurring health care expenditures. Immunization in a community reduces the risk of vaccine-preventable diseases for everyone, including individuals that have not been immunized. In this scenario social benefits exceed private benefits. Thus, the government has a large responsibility in the market of public goods through subsidies, taxes, and regulatory mechanisms [15].

Thirdly, the state has a responsibility to protect both informed and uninformed citizens irrespective of their level of education and income. For example, without government subsidies over half of Africa’s population would not have access to health services. For example, 52 per cent of childbirths are not attended by skilled health personnel [11].

#### General government expenditure on health as a percentage of total government expenditure

**Figure 3:**
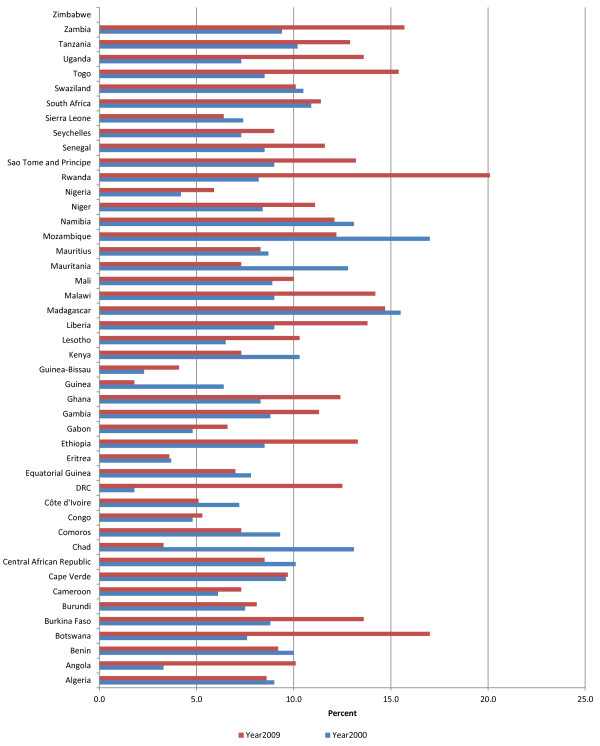
General government expenditure on health as % of total government expenditure.

Figure 3 indicates general government expenditure on health as a percentage of total government expenditure. On average general government expenditure in the AFR was 8.2% of total government expenditure in 2000 and 9.6% 2009. However, there was significant variation across countries. For example, number of countries spending over 10% increased from 10 in 2000 to 23 in 2009. While the number of countries spending over 15% increased from 2 in 2000 to 4 in 2009. About 28 governments increased their expenditures on health and 17 countries registered a decrease between 2000 and 2009. Generally most governments increased their funding for health since the 2001 Abuja Heads of State commitment to target allocating at least 15% of national budgets on health. However, by end of 2009, only four countries (Botswana, Rwanda, Togo and Zambia) had met the Abuja target. It is noteworthy that annual per capita total spending on health for Botswana, Rwanda, Togo and Zambia was US$581, US$52, US$41 and US$63 respectively; which was above the minimum US$34 per capita per year recommended by the WHO Commission for Macroeconomics and Health [4]. However only three of them met the High Level Taskforce on Innovative Financing for Health Systems (HLTIF) [7] recommendation of US$44 per capita per year.

How does the African Region compare with the other five WHO Regions? In terms of average general government expenditure on health as a percentage of total government expenditure AMR was leading with 16.9%, EUR 14.6%, WPR 14.4%, AFR 9.6%, EMR 7.1%, and SEAR 4.9%.

The portion of total government expenditure on health sector depends on factors such as proportion of total national health services provided by government, percentage of population living below poverty line (who will depend on public sector health services), total government revenue (which depends on fees for services provided by government entities, tax-base and tax administration efficiency), the ratio of debt to GDP, burden of debt servicing, size of GDP and its growth, the priority government attaches to health development, and the level of international health funding [16].

#### Social security expenditure on health

The International Social Security Association defines social security as [17]:

“any programme of social protection established by legislation, or any other mandatory arrangement, that provides individuals with a degree of income security when faced with the contingencies of old age, survivorship, incapacity, disability, unemployment or rearing children. It may also offer access to curative or preventive medical care. … social security can include social insurance programmes, social assistance programmes, universal programmes, mutual benefit schemes, national provident funds, and other arrangements that form part of a country’s social security system.”

**Figure 4:**
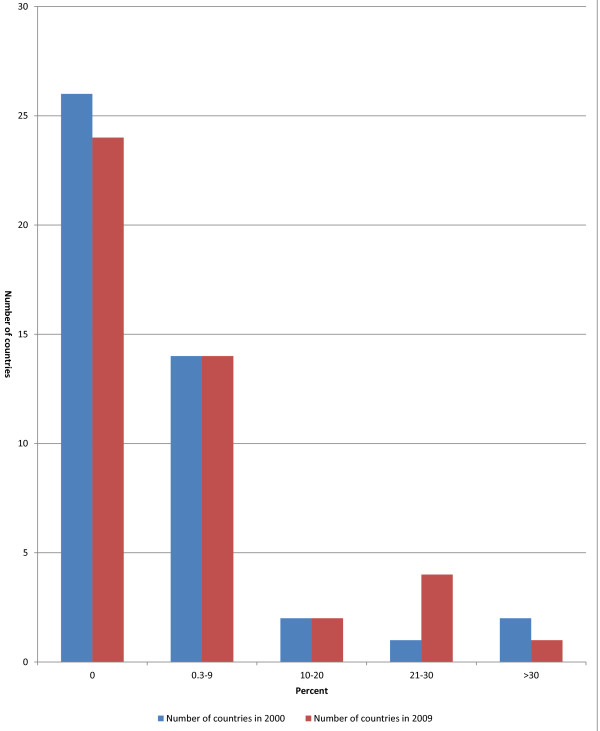
Social security expenditure on health as % of general government expenditure on health.

Figure 4 shows the social security expenditure on health as percentage of general government expenditure on health. Notably, no health expenditures were financed through social security in 26 countries in 2000 compared to 24 countries in 2009. It was only in Algeria, Burundi, Cape Verde, Gabon and Ghana where social security expenditure on health constituted over 20% of general government expenditure on health in 2009.

In AFR average social security expenditure on health was 7.9% and 7.0% of general government expenditure on health in 2000 and 2009 respectively. In 2009 the social security expenditure component of general government expenditure on health was highest in AMR at 72% followed by WPR at 68.6%, EUR 49.5%, EMR 19.4%, SEAR 14.4% and AFR 7.0%.

In 2005 the World Health Assembly adopted resolution WHA58.33 entitled “Sustainable health financing, universal coverage and social health insurance” [18]. The resolution urges Member States:

*“to ensure that health-financing systems include a method for prepayment of financial contributions for health care, with a view to sharing risk among the population and avoiding catastrophic health-care expenditure and impoverishment of individuals as a result of seeking care;….; to plan the transition to universal coverage of their citizens so as to contribute to meeting the needs of the population for health care and improving its quality, to reducing poverty, to attaining internationally agreed development goals, including those contained in the United Nations Millennium Declaration, and to achieving health for all;…”* (p.139).

In 2006 the fifty-sixth session of the WHO Regional Committee for Africa adopted resolution AFR/RC56/R5 entitled ‘Health financing: a strategy for the African Region’ [19]. It urges member states to strengthen the national prepaid health financing systems, including financing structures, processes and management systems.

World Health Report 2010 indicates that universal coverage can be attained through a combination of tax-based funding and social health insurance. Out of 46 countries in the African region, two countries (Rwanda and Ghana) have managed to cover 65% of their population through health insurance [20].

### Private health financing

**Figure 5:**
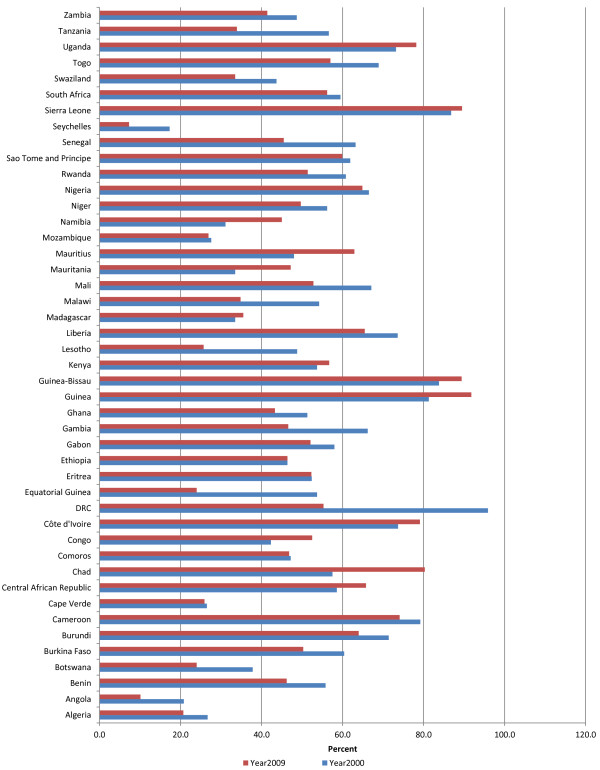
Private expenditure on health as % of total expenditure on health.

In the African Region, private health funds mainly come from household’s out-of-pocket payments and private health insurance that includes community prepayment schemes. Figure 5 shows the private expenditure on health as a percentage of total expenditure on health (PrTHE). Average PrTHE in the African Region was 56.1% in 2000 and 50.7% in 2009. In 2000, private expenditure on health constituted 31-60% in 23 countries compared to 25 countries in 2009.

Average PrTHE was highest in SEAR with 62.9% compared to AFR and AMR with 50.7%, EMR 49.1%, WPR 35.6%, and EUR 24.8%.

#### Household out-of-pocket expenditures

**Figure 6:**
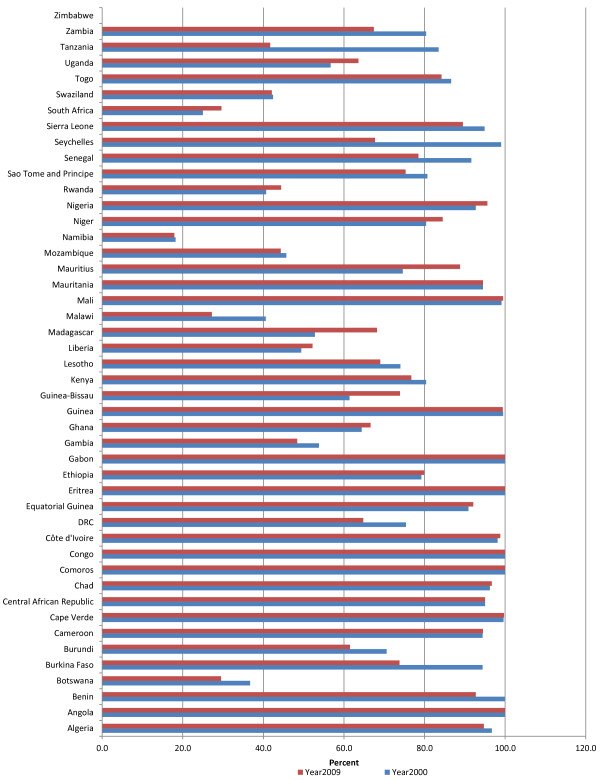
Out-of-pocket expenditure as % of private expenditure on health.

Figure 6 shows household direct out-of-pocket spending (OOPS) on health as a percentage of private expenditure on health. On average OOPS constituted 56.7% and 61.6% of private expenditure on health in 2000 and 2009, respectively. In the African Region over 70% of private expenditure on health came from OOPS in 32 (71%) countries and in 27 (60%) countries in 2000 and 2009, respectively. During the period between 2000 and 2009 seventeen countries recorded an increase in percentage of private expenditure incurred by households through OOPS; twenty one countries had a decrease; and the share remained constant in seven countries.

In 2009 the EMR had the highest average percentage of private expenditure coming household OOPS of 88.2% compared to 84.4% in SEAR, 78.6% in WPR, 69.7% in EUR, 61.6% in AFR and 31.2% in AMR.

Evidence shows that when OOPS as a proportion of THE is below 15-20%, the incidence of financial catastrophe caused by out-of-pocket health expenses is negligible [20]. In 2009, OOPS made up over 20% of THE in 34 AFR countries (76%); and more than 50% in 14 countries. Table 1 compares OOPS as a proportion of THE across the six WHO Regions. In 2009, OOPS as a percentage of THE was over 20% in 27 countries (77%) of AMR; 14 countries of EMR (70%); 31 countries of EUR (58%); 7 countries of SEAR (70%); and 10 countries of WPR (37%).

**Table 1:** Out-of-pocket health expenditure as a proportion of total health expenditure, 2009 (%)

**Region**	**Number of countries**			
	**<=20%**	**21-49%**	**50% and above**	**Total number of countries**
AFR	11	20	14	45
AMR	8	22	5	35
EMR	6	7	7	20
EUR	22	26	5	53
SEAR	3	4	3	10
WPR	17	6	4	27

Note: Data was not available for Zimbabwe in AFR, Somalia in EMR, and Democratic Republic of Korea in SEAR.

In the AFR OOPS consist mainly of household payment of user fees to public, non-governmental, and private health service providers. The proponents expected introduction of OOPS to lead to significant revenues for the health sector, improve adherence to referral system and hence efficiency of national health services, curb excessive/frivolous consumption health services, improve quality of health services, and improve equity [21-23]. Lagarde and Palmer [24] did a Cochrane review of 16 studies that found that when fees were introduced or increased, the use of health services decreased significantly. Only two of the reviewed studies found increases in health service use when quality improvements were introduced at the same time as user fees.

Xu *et al.*[25] analysed household surveys in 59 countries to determine the proportion of households facing catastrophic out-of-pocket health expenses, i.e. households whose financial contribution to the health system exceed 40% of income remaining after subsistence needs have been met. They found that the proportion of households facing catastrophic payments varied widely between countries from less than 0.01% in Czech Republic of Slovakia to 10.5% in Vietnam. The proportion of households facing catastrophic payments among six African Region countries (Ghana, Mauritius, Namibia, Senegal, South Africa and Zambia) included in their 2007 study varied from 0.03% in South Africa to 2.29 in Zambia. Xu *et al.* identified the key preconditions for catastrophic payments: the availability of health services requiring payment, low capacity to pay, and the lack of prepayment or health insurance.

As shown in Table 2, a more recent analysis revealed that the mean household incidence of catastrophic health expenditure (HHICE), among 18 African Region countries, ranged from 4.0% in Namibia to 33.48% in Comoros [26]. A multivariate linear regression of mean HHICE against OOPS as percentage of THE, GINI coefficient, and gross national income per capita (GNIPC) yielded the following slope coefficients 0.165 (t = 1.96, n = 17), 0.195 (t = 1.18, n = 17), and −0.00081 (t = −1.66, n = 17), respectively. Thus, there is a positive relationship between the HHICE and both the OOPS as percentage of THE and the GINI coefficient. However, there is a negative relationship between the HHICE and GNIPC. The adjusted coefficient of determination was 21.54; implying that the three independent variables explained only about 22% of the total variations in HHICE.

**Table 2:** Mean incidence of catastrophic expenditure, OOPS as% of THE, GINI Coefficient and GNI per capita

**Country**	**HHICE**	**OOPS as% of THE**	**GINI coefficient**	**Gross national income per capita (PPP Int.$)**
Burkina Faso	20.20%	37%	39.6	1,160
Chad	12.16%	78%	39.8	1,200
Cote D’Ivoire	17.53%	78%	48.4	1,780
Congo	21.89%	53%	47.3	2,880
Comoros	33.48%	47%	64.3	1,090
Ethiopia	9.35%	37%	29.8	950
Ghana	16.99%	29%	42.8	1,540
Kenya	9.85%	44%	47.7	1,580
Mali	19.14%	53%	39	980
Mauritania	12.23%	45%	39	2,400
Mauritius	8.21%	56%	39	13,180
Malawi	7.15%	10%	39	820
Namibia	4.00%	8%	74.3	6,190
Senegal	16.18%	36%	39.2	1,860
Swaziland	9.57%	10%	50.7	5,580
South Africa	7.32%	17%	57.8	10,060
Zambia	4.59%	28%	50.7	1,410
Zimbabwe	7.19%	-	50.1	500

Note: Incidence of catastrophic expenditures from Saksena *et al.*[26]; OOPS as% of THE from World Health Statistics 2012 [11]; GINI coefficient from UNDP Human Development Report 2009 [27]; and GINI coefficient for Mauritius from CIA World Fact Book [28]; GNI per capita from World Bank databank [29].

OOPS keep the poor in poverty and push the near poor below the poverty line. Leive and Xu [30] undertook a study in 15 countries in the African Region that revealed that on average 30% of all households financed OOPS health expenditures by borrowing and selling assets. The implications of this finding are even dire when viewed in the light of the fact that 52.3% of the population of the African Region live on less than one international dollar a day [11].

Given the growing evidence of the negative effects of OOPS, pressure is mounting on Governments in the Region to abolish user fees. Nevertheless, such policies should be guided by evidence in order to prevent disruption of national health services which are often facing resource gaps. Gilson and McIntyre [31] proposed practical strategies for managing fee removal, which include designating a government unit to coordinate managed fee removal, communicating with managers and health workforce, establishing new funds at local level, mass public information campaign, planning medicines and workforce needs to cope with utilization surge, improving physical access to services, and monitoring utilization trends.

#### Private prepaid plans

**Figure 7:**
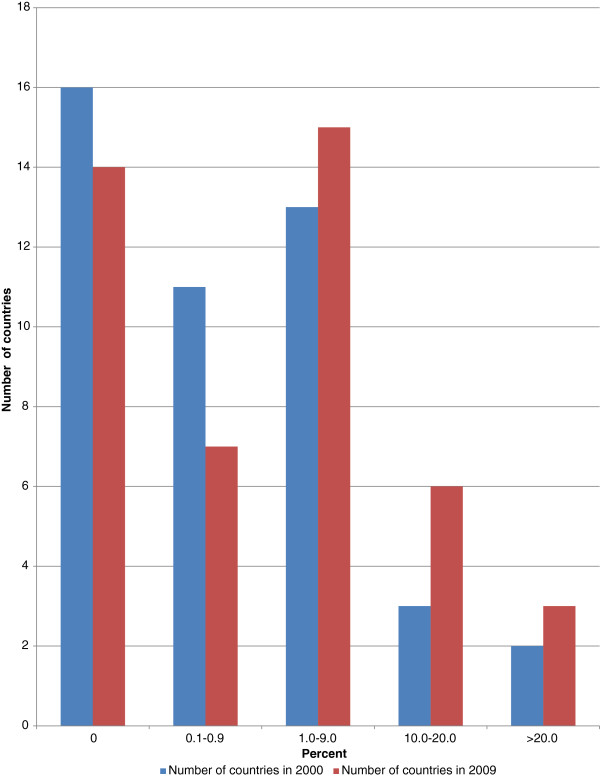
Private prepaid plans as % of private expenditure on health.

Private health insurance or prepaid plans refer to voluntary, risk-rated, for-profit health insurance [32]. Figure 7 portrays private prepaid plans spending on health as a percentage of private expenditure on health. The number of countries where private prepaid plans made some contribution to private health spending increased slightly from 29 in 2000 to 31 in 2009. Seventeen countries witnessed a gradual increase in the contribution of private prepaid plans to private expenditure on health between 2000 and 2009. The increase may partly be attributed to growth in community-based health insurance schemes organized for and/or by workers in the informal economy [33].

The private prepaid plans in AMR made up 61.9% of private expenditure on health; AFR 30.8%; EUR 21.4%; WPR 9.6%; EMR 6.1%; and SEAR 3.5%. However, it is important to recall that the average for the African Region is distorted by the statistics for Namibia and South Africa where private health insurance make up a significant contribution to private expenditure on health. It is important to note that even in Namibia and South Africa, the private health insurance covers relatively small proportions of their populations.

Private health insurance suffers a number of problems [34]. Firstly, there might be adverse selection, when only individuals with a high risk of illness have financial incentive to purchase health insurance. Secondly, there may be moral hazard, over consumption of health care simply because individuals are insured. Thirdly, health service providers might prescribe unnecessary diagnostic tests or perform unnecessary procedures when the patient has health insurance coverage. Fourthly, the chronically sick and the elderly may not be able to afford private health insurance since the premiums are risk-rated. Thus, the private health insurance companies might select, for membership, only people with low risks of demanding health care. This would aggravate inequities in access to health services among different socioeconomic and epidemiological groups of people.

In spite of the potential problems mentioned above, as Sekhri and Savedoff [32] explains that private health insurance can be leveraged for greater good. Firstly, it affords protection from potentially catastrophic health care expenditures to those who can afford to be members. Secondly, although difficult in practice, when adequately regulated, it could be one way of transiting to prepayment and risk-pooling. Thirdly, even in the presence of social health insurance (SHI), private health insurance might be necessary for supplementary coverage for services not in the SHI benefit package [35].

### Rest of the world funds

**Figure 8:**
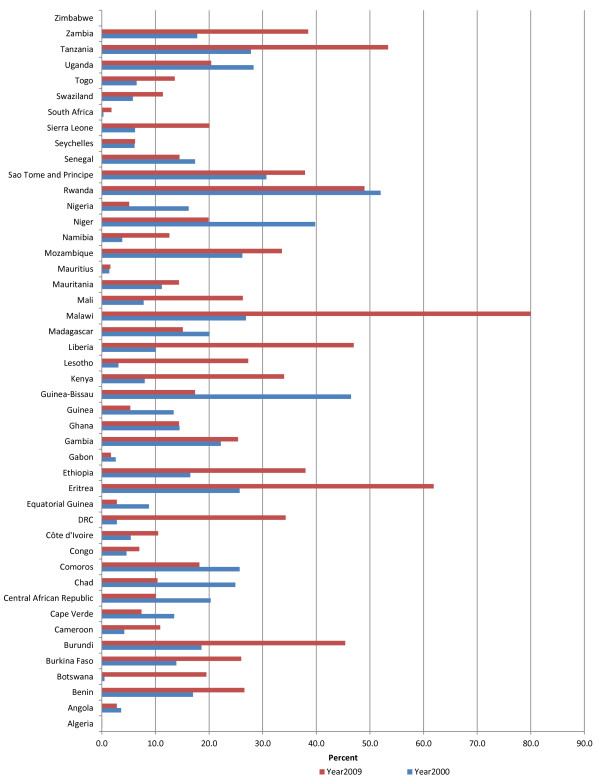
External resources for health as % of total expenditure on health.

Figure 8 shows external resources for health (ERH) as a percentage of total expenditure on health. In 2000, ERH constituted less than 20% of THE in 31 countries; 20-40% in 12 countries; and over 40% in two countries. In 2009, ERH made up less than 20% of total expenditure on health in 26 (58%) of countries; 20-40% in 13 countries; and over 40% in 6 countries. Therefore, 31 (69%) countries and 26 (58%) countries financed less than 20% of their total health expenditure using external resources in 2000 and 2009, respectively.

On average external sources contributed 6.5% and 10.2% of the total expenditure on health in the AFR in 2000 and 2009 respectively. In 2008, AFR was more dependent on external funding for health with an average of 10.2% of THE compared 1.6% in SEAR, 1.1% in EMR, 0.2% in WPR, and 0.1% in EUR and AMR.

Dependence of some African region countries on external sources for financing a significant portion of their total health expenditure is risky for various reasons identified by the Commission for Africa [36]. Firstly, Aid is seldom aligned with recipient countries budget cycles and many donors’ commitments remain unpredictable. Secondly, some donors continue to fund their own priorities which may not be consistent with priorities of recipient countries’. Thirdly, parallel disbursements, reporting, monitoring and review procedures of various donors are time consuming and administratively cumbersome.

In March 2005, the Ministers of donor and developing countries responsible for promoting development and Heads of multilateral and bilateral development institutions adopted the Paris Declaration on Aid Effectiveness aimed at addressing the abovementioned challenges, among others [37]. In the declaration, there is an agreement that recipient countries exercise effective leadership over their development policies and strategies, and coordinate development actions; donors align their support with recipient countries’ national development strategies, institutions and procedures; donors ensure their actions are more harmonized, transparent and collectively effective; managing resources and improving decision-making for results; and mutual accountability for development results. In 2006, the WHO Regional Office for Africa initiated the creation of Harmonization for Health in Africa (HHA), which initially involved international financing institutions (African Development Bank, World Bank) and United Nations Agencies (UNICEF, UNFPA, UNAIDS) with the purpose of facilitating the implementation of Rome and Paris Declarations aspects of external aid coordination in the health sector [38]. In September 2008, at meeting in Accra (Ghana), the donor and developing countries adopted the Accra Agenda for Action which acknowledges inadequate progress and stipulates actions needed to accelerate the pace of change [39].

### Selected per capita indicators for expenditure on health

#### Per capita total expenditure on health

**Figure 9:**
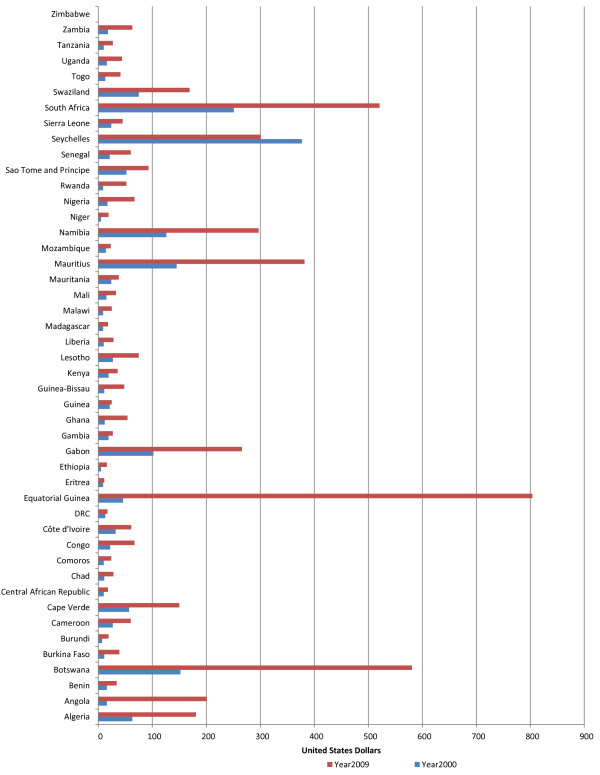
Per capita total expenditure on health at average exchange rate (US$).

Figure 9 presents per capita total expenditure on health. In 2000, per capita total expenditure on health per year was less than US$ 44 in 34 countries; between US$ 44 and US$ 60 in three countries; and over US$60 in eight countries. In 2009, per capita total expenditure on health was less than US$44 in 21 countries; US$44-US$60 in 7 countries; and over US$60 in 17 countries. The per capita total expenditure on health varied from a minimum of US$11 to a maximum of US$804 [11].

The 2001 WHO Commission of Macroeconomics and Health (CMH) estimated that US$34 per person was needed to provide a package of essential health services [4]. Between 2000 and 2009 the number of countries achieving the CMH recommendation increased from 11 to 29.

In 2009, the HLTIF estimated that on average US$44 per capita would be needed to strengthen health systems as well as provide essential services in 49 low-income countries [7]. The number of countries allocating US$44 per person per year in AFR increased from 11 (24%) in 2000 to 24 (53%) in 2009. In fact, except one country which registered a 20% decrease, all the other 44 countries in the AFR recorded some nominal growth in per capita total expenditure on health between 2000 and 2009. The percentage growth between 2000 and 2009 varied significantly between 19% and 91% in eleven countries; 100-200% in twenty countries; and above 200% thirteen countries. Notably, Angola and Equatorial Guinea registered a percentage growth in per capita total expenditure on health of over 1000%.

The AFR average per capita total expenditure on health was US$35 in 2000 and US$82 in 2009. In 2009 AFR average per capita total expenditure was 1.7 fold higher than that of SEAR, but 39-fold lower than AMR, 27-fold lower than EUR, 6-fold lower than WPR, and 2-fold lower than EMR. Between 2000 and 2009, SEAR registered the highest percentage growth in per capita total spending on health of 153%, EUR 135%, AFR 134%, EMR 88%, WPR 79% and AMR 72%.

#### Per capita government expenditure on health

**Figure 10:**
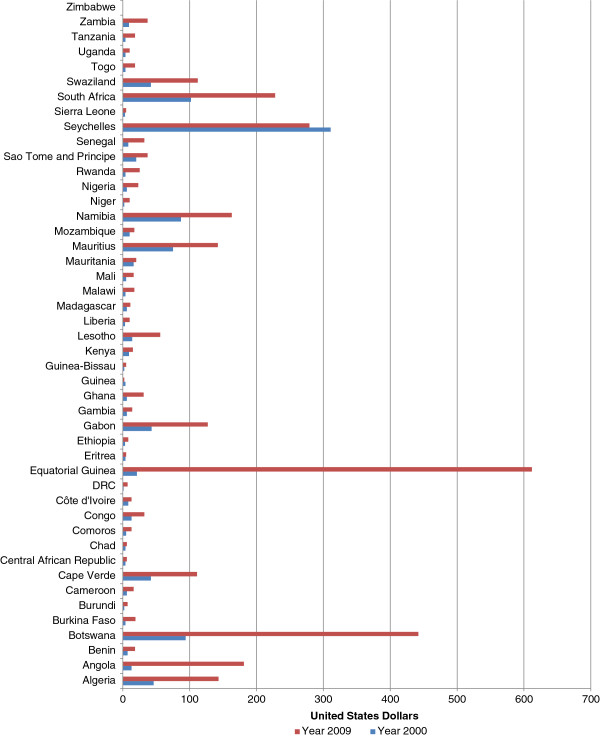
Per capita government expenditure on health at average exchange rate (US$).

Figure 10 shows per capita government expenditure on health. In 2000, the government expenditure on health per person per year was less than US$10 in twenty-nine countries; between US$ 10 and US$ 30 in seven countries; and over US$ 30 in nine countries. In 2009 per capita government expenditure on health was less than US$10 in 9 countries; between US$10 and US$30 in 19 countries; and over US$30 in 17 countries. It varied from a minimum of US$2 to a maximum of US$612. Fifty-six per cent of the countries in the region had a per capita government expenditure on health of less than US$20.

Two countries recorded a negative growth in per capita government expenditure on health of 50% and 10%, respectively. On the other hand, all the other countries reported a positive growth in per capita government expenditure on health varying between 25% and 89% in 12 countries; between 100 and 200% in 12 countries; and over 200% in 19 countries.

In AFR the per capita government expenditure on health increased from US$15 in 2000 to US$41 in 2009. The AFR per capita government expenditure on health in 2009 was 2-fold higher than that of SEAR. However, AFR per capita government expenditure on health was 41-fold lower than EUR, 38-fold lower than AMR, 9-fold lower than WPR and 2-fold lower than EMR.

In terms of growth in per capita government expenditure SEAR was leading with 217% followed by AFR 173%, EUR 138%, EMR 113%, AMR 89% and WPR 72%.

### Limitations of the study

(a). A sizeable number of countries in the AFR have not conducted even a single round of NHA, and thus, the estimates contained in the World Health Statistics 2012 are collated from different sources. Therefore, even though those are the best estimates available internationally, we do not know to what extent such estimates suffer the data source weaknesses discussed in Chapter 6 of the World Health Organization, World Bank and USAID Guide to producing national health accounts [10].

(b). The NHA data contained in the World Health Statistics 2012 report does not address allocations of health expenditures per public health functions/programmes and health system components (this could reveal distortions in budget allocations, not consistent with national health policies and priorities). It does not show allocations from central, intermediate and local levels; allocations by different group of populations, but rather averages that may hide serious inequities.

## Conclusion

Between years 2000 and 2009, in the African Region: (a) the percentage of GDP spent on health increased in 34 countries, decreased in 10 countries, and remained constant in 1 country; (b) government spending on health as a percentage of THE increased in 31 countries and decreased in 13 countries; (c) private prepaid plans made some contribution to private health spending in to 31 countries; (d) number of countries meeting the Abuja target increased from 2 to 4 countries; (e) number of countries financing more than 20% of their THE from external sources increased from 14 to 19; (f) number of countries spending at least US$44 per person per year, in line with the recommendation of the HLTIF, increased from 11 (24%) to 24 (53%) countries; (g) 43 (95%) of the countries reported a positive growth in per capita government expenditure on health varying between 25% and 2814%. In addition, in 2009, OOPS made up over 20% of total expenditure on health in 34 countries.

It is evident that while the AFR average government expenditure on health as a per cent of THE increased by 5.4 per cent, the average PrTHE decreased by the same percentage between 2000 and 2009. However, average OOPS as a per cent of PrTHE increased by 4.9 per cent. The average ERH as a percentage of THE increased by 3.7 per cent.

On balance, has the status of health care financing improved? The objectives of health financing system are to raise sufficient funds, protect people from the financial consequences of ill-health and paying for health services, and improve efficiency and equity. Judgement of whether health care financing has improved between 2000 and 2009 requires an assessment of the extent to which those objectives have been realized. There is evidence that per capita total expenditure on health increased from US$35 in 2000 to US$82 in 2009 in the AFR. However, the fact that OOPS as a percentage of THE was above the threshold of 20% in 34 countries, suggests that significant sections of populations in those countries have no protection from risk of financial catastrophe. Also even though measurement of efficiency of health systems was beyond the scope of this study, there is information in the World Health Report 2010 that approximately 20-40% of health resources are being wasted [20]. Therefore, even though on average the quantity of health funds have increased, we cannot judge from the current study the extent to which financial risk protection, equity and efficiency has progressed or regressed.

Evidence that OOPS as a percentage of THE is above the 20% catastrophic threshold in over 70% of countries in the AFR implies that there is pressing need for countries to develop health policies that address inequities and health financing models that optimize the use of health resources and strengthen health infrastructure. In addition, it means that there is need to increase coverage of prepaid health-financing mechanisms to reduce over-reliance on potentially catastrophic and impoverishing out-of-pocket payments [20].

## Abbreviations

AFR: WHO African Region; AMR: WHO Region of the Americas; CMH: WHO Commission of Macroeconomics and Health; EMR: WHO Eastern Mediterranean Region; ERH: External resources for health; EUR: WHO European Region; GDP: Gross domestic product; GNI: Gross national income; GNIPC: Gross national income per capita; GNP: Gross national product; HHA: Harmonization for Health in Africa; HHIC: Household mean incidence of catastrophic expenditure; HLTIF: High Level Taskforce on Innovative Financing for Health Systems; OOPS: Out-of-pocket spending; PrTHE: Private expenditure on health as a percentage of total expenditure on health; SEAR: WHO South-Eastern Asia Region; SHI: Social health insurance; SSA: Sub-Saharan Africa; THE: Total expenditure on health; UNAIDS: Joint United Nations Programme on HIV and AIDS; UNFPA: United Nations Population Fund (formerly known as United Nations Fund for Population Activities); UNICEF: United Nations International Children’s Emergency Fund; WHO: World Health Organization; WPR: WHO Western Pacific Region

## Competing interests

The authors declare that they have no competing interests.

## Authors’ contributions

LGS, JMK, and JNO contributed to the design, literature review, analysis and writing of various sections of the manuscript. All authors read and approved the final manuscript.

## References

[B1] United Nations United Nations Millennium Declaration 2000 New York: United Nations

[B2] Organization of African Unity Abuja declaration on HIV/AIDS, tuberculosis and other related infectious diseases Decision OAU/SPS/Abuja/3 2001 Addis Ababa: Organization of African Unity

[B3] United Nations Report of the International Conference on Financing for Development Monterrey, Mexico, 18–22 March 2002 2002 New York: United Nations

[B4] Macroeconomics and Health Investing in health for economic development — Report of the Commission on Macroeconomics and Health 2001 Geneva: WHO

[B5] United Nations International Bill of Human Rights: a universal declaration of human rights 1948 New York: United Nations

[B6] WHO Ouagadougou declaration on Primary Health Care and health systems in Africa: achieving better health outcomes for Africa in the new millennium 2008 Brazzaville: WHO/AFRO

[B7] Taskforce on Innovative International Financing for Health Systems Constraints to scaling up and costs: working group 1 report 2009 Geneva: International Health Partnership

[B8] Taskforce on Innovative International Financing for Health Systems More money for health, and more health for the money: final report 2009 Geneva: International Health Partnership

[B9] World Health Organization World Health Statistics 2011 2011 Geneva: WHO

[B10] World Health Organization WHO, World Bank and USAID: Guide to producing national health accounts; with special application for low-income and middle-income countries 2003 Geneva: WHO

[B11] World Health Organization World Health Statistics 2012 2012 Geneva: WHO

[B12] Kirigia JM Mwikisa CN Nganda BM Cardoso B Effects of global financial crisis on funding for health development in nineteen countries of the WHO African region BMC Int Health Hum Right 2011 11 4 URL: http://www.biomedcentral.com/1472-698X/11/4 10.1186/1472-698X-11-4 PMC3094291 21489284

[B13] Zacher MW Kaul I, Grunberg I, Stern MA Global surveillance: international cooperation to monitor infectious diseases Global public goods: international cooperation in the 21st century 1999 Oxford: Oxford University Press 266 283

[B14] Kaul I Grunberg I Stern MA Kaul I, Grunberg I, Stern MA Defining global public goods Global public goods: international cooperation in the 21st century 1999 Oxford: Oxford University Press 2 19

[B15] Skaggs NT Carlson JL Microeconomics 1996 Oxford: Blackwell Publishers Ltd

[B16] Musgrave RA Musgrave PB Public finance in theory and practice 1982 London: McGraw-Hill

[B17] International Social Security Associationwebpage http://www.issa.int/ Accessed at 14 h00 on 4^th^ February 2013

[B18] WHO Fifty-eighth World Health Assembly resolutions and decisions 2005 Geneva: WHO

[B19] WHO Health financing: a strategy for the African Region 2006 Brazzaville: WHO/AFRO

[B20] WHO World Health Report 2010 – Health systems financing: the path to universal coverage 2010 Geneva: WHO 10.2471/BLT.10.078741 PMC2878164 20539847

[B21] World Bank Financing the health sector: an agenda for reform 1987 Washington, DC: The World Bank

[B22] McPake B User charges for health services in developing countries: a review of the economic literature Soc Sci Med 1993 36 11 1397 1405 10.1016/0277-9536(93)90382-E 8511628

[B23] Lagarde M Palmer N The impact of user fees on access to health services in low- and middle income countries The Cochrane Library 2011 4 1 68 10.1002/14651858.CD009094 PMC10025428 21491414

[B24] Lagarde M Palmer N The impact of user fees on health service utilization in low- and middle-income countries: how strong is the evidence? Bull World Health Organ 2008 86 11 839 851 10.2471/BLT.07.049197 19030689 PMC2649541

[B25] Xu K Evans DB Kawabata K Zeramdini R Klavus J Murray CJL Household catastrophic health expenditure: a multi-country analysis Lancet 2003 362 111 117 10.1016/S0140-6736(03)13861-5 12867110

[B26] Saksena P Xu K Durairaj V The drivers of catastrophic expenditure: outpatient services, hospitalization or medicines? World Health Report 2010 Background Paper 21 2010 Geneva: WHO

[B27] UNDP Human development report 2009 2009 New York: Oxford University Press

[B28] United States Government CIA World Factbook 2009 Washington, D.C.: Central Intelligence Agency

[B29] World Bankhttp://databank.worldbank.org/ddp/home.do? Accessed at 16h 30 on 5 February 2013

[B30] Leive A Xu K Coping with out-of-pocket health payments: empirical evidence from 15 countries Bull World Health Organ 2008 86 11 849 856 10.2471/BLT.07.049403 19030690 PMC2649544

[B31] Gilson L McIntyre D Removing user fees for primary care in Africa: the need for careful action Br Med J 2005 331 762 765 10.1136/bmj.331.7519.762 16195296 PMC1239983

[B32] Sekhri N Savedoff W Private health insurance: implications for developing countries Bull World Health Organ 2005 83 2 127 134 15744405 PMC2623814

[B33] Ginneken W Audibert M, Mathonnat J, Roodenbeke E Health insurance for workers in the informal economy: exploring the potential of micro-insurance schemes. Chapter 11 Le financement de la sante dans les pays d’Afrique et d’Asie a faible revenue 2003 Paris: Karthala

[B34] Morris S Health economics for nurses 1998 London: Prentice Hall

[B35] Evans DB Carrin G Evans TG The challenge of private insurance for public good Bull World Health Organ 2005 83 2 83 15744395 PMC2623807

[B36] Commission for Africa Our Common Interest – report of the Commission for Africa 2005 London: Commission for Africa

[B37] OECD The Paris declaration on aid effectiveness 2005 Paris: OECD

[B38] Harmonization for Health in Africa Operational guide 2010 Brazzaville: WHO/AFRO

[B39] OECD Accra Agenda for Action 2008 Paris: OECD

